# Lymphogranuloma venereum (LGV) in men who have sex with men (MSM): a re-emerging problem, Malta, 2018

**DOI:** 10.2807/1560-7917.ES.2018.23.43.1800541

**Published:** 2018-10-25

**Authors:** Alastair Donachie, Gianfranco Spiteri, Christopher Barbara, Tanya Melillo, Ronza Hadad, Alexandra Gauci Farrugia, Magnus Unemo, Valeska Padovese

**Affiliations:** 1 Infectious Disease Prevention and Control Unit (IDCU) – Health Promotion and Disease Prevention Directorate, Valletta, Malta; 2 European Programme for Intervention Epidemiology Training (EPIET), European Centre for Disease Prevention and Control (ECDC), Stockholm, Sweden; 3 These authors contributed equally to the work; 4 European Centre for Disease Prevention and Control (ECDC), Stockholm, Sweden; 5 Department of Pathology, Mater Dei Hospital, Msida, Malta; 6 WHO Collaborating Centre for Gonorrhoea and Other STIs, National Reference Laboratory for STIs, Faculty of Medicine and Health, Örebro University, Örebro, Sweden; 7 Genitourinary Clinic, Mater Dei Hospital, Msida, Malta

**Keywords:** LGV, MSM, Malta, Lymphogranuloma venereum, men who have sex with men, outbreak, group sex

## Abstract

From 1 January to 30 June 2018, 11 cases of Lymphogranuloma venereum (LGV; all preserved samples (n = 4) genovar L2b) were identified at the Genitourinary Clinic (GUC), Mater Dei Hospital, Msida, Malta. All cases were diagnosed in men who have sex with men (MSM); six participated in three group-sex parties. Here, we describe the outbreak and risk factors associated with LGV diagnoses in MSM in Malta in 2018.

In March 2018, four men who have sex with men (MSM) sought care at the Genitourinary Clinic (GUC) at Mater Dei Hospital, Msida, Malta as contacts of *Chlamydia trachomatis;* patients that tested positive for *C. trachomatis* were subsequently tested for Lymphogranuloma venereum (LGV).

## Laboratory testing at the Genitourinary Clinic and LGV treatment

Based on sexual history and orientation clients attending GUC are offered nucleic acid amplification tests (NAATs; AmpliSens 4-plex for detection of *Chlamydia trachomatis* (Ct), *Neisseria gonorrhoeae* (Ng), *Mycoplasma genitalium* (Mg) and *Trichomonas vaginalis* (Tv) -multiprime-FRT) from urine, vaginal, throat and rectal samples which are routinely performed at the clinic’s laboratory. Serological tests for HIV, hepatitis B and C, and syphilis are also offered (clients can choose to opt out).

Since April 2016, all Ct-positive rectal swabs from MSM have been sent to a commercial laboratory in Germany for detection of genovars L1-L3, irrespective of HIV status and the presence of anorectal symptoms, as recommended by the 2015 European Ct guideline [1]. All Ct-positive cases were treated with the recommended doxycycline 100 mg twice daily for 7 days and in case of positive L genovars, treatment was continued up to 21 days [1-3]. The test of cure (TOC) for Ct on urine and rectal samples was performed ≥21 days post-treatment. Preserved LGV-positive samples were further genotyped using *ompA* sequencing, as previously described [4]. Contact tracing was conducted for all confirmed LGV cases. Sexual contacts and partners were advised to visit the GUC for testing.

## Outbreak investigation

**Figure 1:**
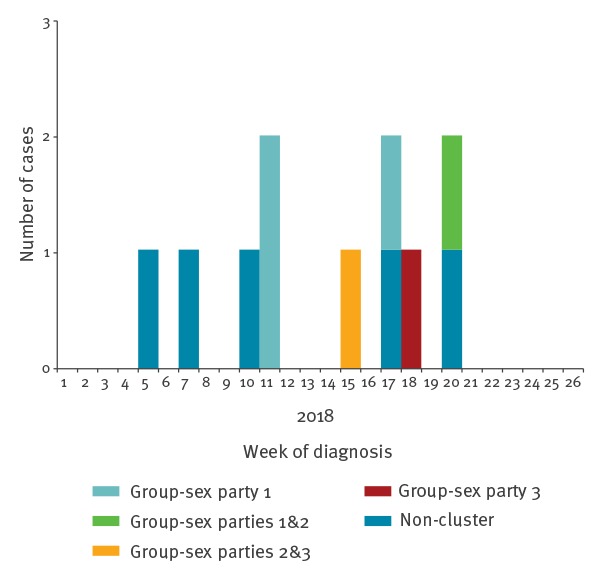
Epidemic curve of Lymphogranuloma venereum cases among men who have sex with men, by week of diagnosis, February–May 2018, Malta (n = 11)

From 1 January to 30 June 2018, 40 Ct-positive rectal samples were examined for L1-L3 genovars and 11 (28%) were positive (Figure 1). In 2017, 48 rectal Ct infections were detected among MSM and two (4.2%) of these had confirmed LGV infection. Prior to 2017, only one LGV case was diagnosed in Malta; an HIV-positive MSM in 2013, who was also one of the two cases confirmed in 2017. All cases before 2018 were acquired abroad, two cases were epidemiologically linked to LGV positive contacts in the United Kingdom (UK) in 2013 and the Netherlands in 2017, respectively, and the third case likely acquired the infection during a gay cruise in Spain in 2017.

## Group-sex cases

**Figure 2:**
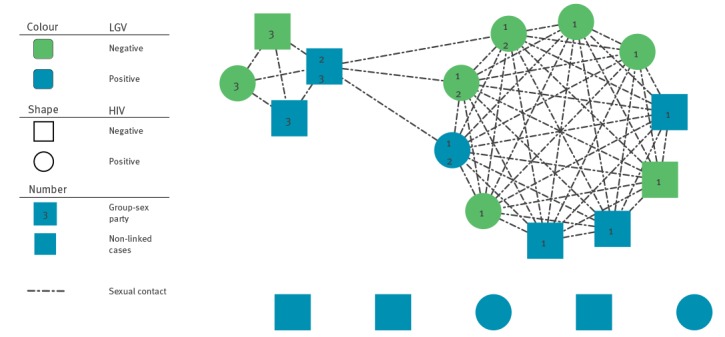
Sexual network diagram of Lymphogranuloma venereum cases among men who have sex with men and their identified contacts, 2018, Malta (n = 19)

Of the 11 LGV cases diagnosed in 2018, six were involved in three private MSM group-sex parties in March 2018. Four LGV cases were linked to party 1, two cases to party 2 and two cases to party 3. One case took part in party 1 and 2 and another case to party 2 and 3. (Figure 2). The parties were organised via MSM social network mobile phone applications. All six cases engaged in group sex and condom-less sex (CLS) and five cases additionally reported to using drugs before engaging in group sex (chemsex), including Gamma Hydroxybutyrate (GHB) and cocaine. Dates of symptom onset of group-sex cases were from 12 March to 16 May 2018 and age range was 21–62 years with a median age of 36 years. Four cases were Maltese (Table).

**Table:** Demographic characteristics, co-infections, risk behaviours and laboratory results for confirmed Lymphogranuloma venereum cases, Malta, 2018

	Case number										
	1	2	3	4	5	6	7	8	9	10	11
Genovar	NA	NA	NA	a	L2b	NA	NA	NA	NA	L2b	L2b
Party	1	1	1	1,2	2,3	3	No	No	No	No	No
Symptom onset month	March	March	April	May	April	May	February	March	February	April	May
HIV status	neg	neg	neg	pos	neg	neg	pos	neg	neg	pos	neg
ART	NA	NA	NA	Not started	NA	NA	Y	NA	NA	Not started	NA
PrEP	Y	N	N	N	N	Y	NA	Y	N	N	Y
STI co-infection	rectal gonorrhoea	rectal gonorrhoea	No	HIV^a^	No	No	No	rectal gonorrhoea	No	rectal gonorrhoea, HIV^a^	No
No. sexual partners (past 3 months)	>10	3	9	>10	6	4	3	>10	2	10	>10
Risky sexual behaviour (Y/N)^b^	Y	Y	Y	Y	Y	Y	Y	Y	Y	Y	Y

ART: antiretroviral treatment; HIV: human immunodeficiency virus; N: no; NA: not applicable; neg: negative; pos: positive; PrEP: pre-exposure prophylaxis; STI: sexually transmitted infection; Y: yes.

^a^Newly diagnosed with HIV at the time of Lymphogranuloma venereum diagnosis.

^b^Recent unprotected intercourse with unknown partners, foreigners or with multiple partners.

All 11 cases were rectal LGV positive, except one with positive genital LGV infection. Of the six group-sex cases, two presented with bleeding per rectum, anal fissure and discharge. Two cases were co-infected with rectal gonorrhoea and one case was also diagnosed with HIV infection concurrently with LGV infection; the other LGV group-sex cases were HIV-negative and two were on HIV pre-exposure prophylaxis (PrEP).

In the 3 months before LGV symptom onset, the six group-sex cases reported a range of 3­-well over 10 sexual partners (median 7.5). One case, who was on PrEP, developed rectal bleeding and discharge in May when travelling in the UK where he was tested and diagnosed with rectal and throat LGV. He was treated empirically with doxycycline 100 mg twice a day for 21 days, ceftriaxone 500 mg intramuscularly and azithromycin 1 g oral dose. Preserved rectal samples from one case (involved in party 1) and second case (involved in party 2 and 3) contained the Ct genovar L2b. Test of cure was negative for all cases after treatment.

A total of eight additional MSM contacts were identified who attended these group-sex parties, two of whom attended party 1 and 2. One MSM contact was rectal Ct-positive, but LGV-negative. Five contacts were known to be HIV-positive and undergoing antiretroviral treatment (ART), one contact was diagnosed with HIV following group sex and two were HIV-seronegative. In addition, one contact was diagnosed with rectal gonorrhoea and two with latent syphilis infection.

## Cases not linked to the group-sex parties

In 2018, five additional LGV rectal cases were confirmed at the GUC, but were not epidemiologically linked to the group-sex parties (Table). Their ages ranged from 21–61 years. Four cases developed symptoms including rectal discharge (n = 3), rectal bleeding (n = 2), constipation (n = 1) and anal fissure (n = 1); one case was asymptomatic.

Three cases were HIV-negative, two of which were likely infected abroad since they did not report having sex while in Malta. Two of the HIV-negative cases were on PrEP and had engaged in CLS but not chemsex or group sex. One asymptomatic case presented at the GUC for sexually transmitted infections (STIs) screening and besides testing rectal LGV positive, he was also Ct positive in urine. One case was diagnosed with rectal gonorrhoea. Test of cures were negative.

Two cases were HIV positive; one case had an undetectable viral load and was receiving ART (Lamivudine, Tenofovir and Kaletra) at the time of LGV diagnosis. He reported engaging in CLS and chemsex as part of group sex at the end of January 2018 in Malta. The other case, an Asian man in his early twenties who was newly diagnosed with HIV and co-infected with rectal gonorrhoea, had engaged in CLS but denied drug use and group sex. A TOC was not possible since the patient had already left the country. Preserved rectal samples from two of the non-linked LGV cases also contained the Ct genovar L2b.

## Discussion

The detection of the first cluster (Ct genovar L2b) and overall increase in LGV diagnoses among MSM in Malta in 2018, coincides with upward trends in other STIs among MSM such as chlamydia, gonorrhoea and syphilis (unpublished data). LGV is a re-emerging STI caused by the invasive Ct L serovars/genovars L1, L2 and L3 [5]. Since 2003, when the first outbreak of LGV among MSM in Europe was reported in the Netherlands, several outbreaks have been documented in Western European countries [6-8]. Cases of LGV are typically notified among HIV-positive MSM who report unprotected anal intercourse, STI co-infection and other high-risk behaviours. In some settings, 25% of LGV infections can be asymptomatic [9].

The Maltese islands are an easy-to-reach and affordable tourist destination, with a flourishing economy that has attracted thousands of workers from all over the world [10]. Gender identity and intersex protection laws in Malta are of high standard and recent changes in legislation saw the Maltese islands rise to top on the International Lesbian, Gay, Bisexual, Trans and Intersex Association (ILGA)-Europe Rainbow Index in 2016 [11]. This has contributed to make the Maltese Islands an increasingly popular travel destination for MSM. Contributing factors to the increase in LGV and other STIs could be due to high-risk behaviours such as high rates of partner change (including new and unknown contacts) facilitated by the use of dating applications, frequent STI co-infections, CLS, group sex and chemsex. Moreover, concern has emerged about how users of biomedical interventions, particularly Treatment as Prevention (TasP) and PrEP, may change their HIV sexual risk behaviours [12-14]. To date, the most comprehensive behavioural study involving MSM living in Malta was from the 2010 European Men-Who-Have-Sex-With-Men Internet Survey (EMIS) which included 123 MSM residing in Malta. However, new data from EMIS 2017 should help us to identify and better understand the sexual knowledge, attitudes, and behaviours contributing to the increase in LGV and other STIs among MSM in Malta [15].

Clinicians should maintain a high index of suspicion for LGV in MSM with rectal symptoms, particularly those who are HIV-positive or are co-infected with other STIs. Sexual contacts of LGV cases should be traced and tested for Ct and presumptive treatment with doxycycline, including of those with urine and throat infections should also be considered [16]. Furthermore, although European guidelines recommend the use of either doxycycline or azithromycin for treatment of urogenital or pharyngeal Ct infection [17], we suggest that MSM positive for Ct in pharynx and/or urine should ideally be treated with doxycycline. Starting or continuing PrEP in HIV-seronegative MSM engaging in high-risk behaviours is also recommended [18].

## Conclusion

A strong public health response to raise awareness of LGV among clinicians, laboratories and MSM in Malta and internationally is essential. This study highlights the importance of partner notification and testing of HIV-positive and/or other STI co-infected symptomatic and asymptomatic MSM for Ct and LGV. Finally, capacity to conduct genotyping to identify serovars/genovar L1, L2 and L3 in Ct-positive samples is necessary in order to ensure rapid diagnosis and subsequent appropriate treatment.
